# Effects of Obesity and Gastric Bypass Surgery on Nutrient Sensors, Endocrine Cells, and Mucosal Innervation of the Mouse Colon

**DOI:** 10.3390/nu10101529

**Published:** 2018-10-17

**Authors:** Madusha Peiris, Rubina Aktar, Sarah Raynel, Zheng Hao, Michael B. Mumphrey, Hans-Rudolf Berthoud, L. Ashley Blackshaw

**Affiliations:** 1 Centre for Neuroscience & Trauma, Blizard Institute, Barts and the London School of Medicine and Dentistry, Queen Mary University of London, London E1 2AT, UK; r.aktar@qmul.ac.uk (R.A.); sarahraynel@hotmail.com (S.R.); a.blackshaw@qmul.ac.uk (L.A.B.); 2 Neurobiology of Nutrition & Metabolism Department, Pennington Biomedical Research Center, Louisiana State University System, Baton Rouge, LA 70808, USA; zheng.hao@pbrc.edu (Z.H.); michael.mumphrey@pbrc.edu (M.B.M.); hans.berthoud@pbrc.edu (H.-R.B.)

**Keywords:** enteroendocrine cells (EECs), appetite regulation, Roux-en-Y gastric bypass (RYGB), weight loss

## Abstract

Background: Nutrient-sensing receptors located on enteroendocrine (EEC) cells modulate appetite via detection of luminal contents. Colonic ‘tasting’ of luminal contents may influence changes to appetite observed in obesity and after weight loss induced by bariatric surgery. We assessed the effects of obesity and gastric bypass-induced weight loss on expression of nutrient-sensing G-protein coupled receptors (GPCRs), EEC and enterochromaffin (EC) cells and mucosal innervation. Methods: qPCR and immunohistochemistry were used to study colonic tissue from (a) chow-fed/lean, (b) high-fat fed/obese, (c) Roux-en-Y gastric bypass surgery (RYGB), and (d) calorie restriction-induced weight loss mice. Results: Expression of GPR41, GPR43, GPR40, GPR120, GPR84, GPR119, GPR93 and T1R3 was increased in obese mice. Obesity-induced overexpression of GPR41, 40, 84, and 119 further increased after RYGB whereas GPR120 and T1R3 decreased. RYGB increased TGR5 expression. L-cells, but not EC cells, were increased after RYGB. No differences in mucosal innervation by protein gene product (PGP) 9.5 and GLP-1R-positive nerve fibers were observed. Stimulation of colonic mucosa with GPR41, GPR40, GPR85, GPR119, and TGR5 agonists increased cell activation marker expression. Conclusions: Several nutrient-sensing receptors induced activation of colonic EEC. Profound adaptive changes to the expression of these receptors occur in response to diet and weight loss induced by RYGB or calorie restriction.

## 1. Introduction

We have previously shown that nutrient-sensing g-protein coupled receptors (GPCRs) are found throughout the gastrointestinal tract (GIT), with colonic expression concentration amongst the highest [1]. Nutrient-sensing GPCRs respond to luminal dietary contents including amino acids (AAs), peptides, short-chain fatty acids (SCFAs), medium-chain fatty acids (MCFAs), long-chain fatty acids (LCFAs), lipid metabolites, and bile acids [2]. Importantly, many of these receptors, including GPR41 and GPR43 (SCFA sensors), GPR84 and GPR40 (MCFA sensors), GPR120 (MCFA/LCFA), and GPR119 (lipid metabolite sensors), are expressed on L-cells and enterochromaffin (EC) cells. Indeed, activation of human colonic GPCRs, such as calcium-sensing receptors (CaSR) and GPR84, induce the release of 5-HT from EC cells, and glucagon-like peptide (GLP-1) and PYY from L-cells [1]. Therefore, the activation of enteroendocrine (EEC) and EC cells is dependent on the expression and ligand-induced activation of nutrient-sensing GPCRs. We explored the concept that changes in the luminal content, e.g., normal diet vs. high-fat vs. undigested nutrients (shunted to the colon as in RYGB), may dynamically alter the type and number of GPCRs, which alters the function of the cells on which they are expressed.

Roux-en-Y gastric bypass (RYGB) is a form of bariatric surgery that has a high level of success with patients consistently losing weight, normalizing insulin sensitivity, and changing eating habits [3,4,5]. These are complex and multifactorial changes that are yet to be completely understood at the molecular level. However, clinical studies have shown that significantly increased postprandial circulation levels of the anorectic and incretin hormones PYY and GLP-1 are likely to be important factors in appetite reduction following RYGB [6]. L-cells are expressed throughout the gut with the highest density found in the colon [7,8]. Therefore, enteroendocrine (EEC) cells in the colon, such as L-cells, are likely important contributors to decreased appetite and food intake following RYGB. This may reflect the ability of the cell to respond to the luminal environment and to sense specific nutrients. In obesity, overall L-cell number decreases, as does the ability of these cells to release GLP-1 [9], suggesting a high-fat diet reduces cell activation. Additionally, 5-HT released from EC cells is likely to have an important role in modulating appetite as nutrients stimulate the release of 5-HT to activate duodenal vagal afferent fibers [10]. Therefore, colonic 5-HT release stimulated by dietary nutrients may have a similar effect on colonic vagal afferents which act on 5-HT3 receptors [11]. Collectively, the activation and release of hormones/transmitters from L-cells and EC cells are critical pathways, and thus it is important to elucidate the mechanisms by which these cells are activated after RYGB treatment.

The aims of this study were to specifically characterize changes to colonic mucosa resulting from exposure to changing luminal contents from a normal nutrient load to a high-calorific load, and to the arrival of undigested food shunted to colon after RYGB treatment. Changes to mRNA expression of nutrient-sensing GPCRs and mediator hormones/peptides, EECs, EC cell population, and neuronal innervation were assessed before a nutritional challenge was given to naïve colons of normal-weight mice to observe the effect on the activation of intracellular pathways. The results of this study will have relevance for the development of non-surgical, luminally-acting treatments for obesity.

## 2. Materials and Methods

### 2.1. Animals

Male C57/BL6J mice were purchased from Jackson Laboratories and individually housed at the Pennington Biomedical Research Center Vivarium in wire mesh cages in climate-controlled rooms (21–23 °C, lights on at 07:00 h, lights off at 19:00 h). Mice were exposed to a high-fat diet (60% energy from fat, Research Diets # 12492) for 12–14 weeks before and 12 weeks after surgery (see below), when tissues were harvested. Animals of the weight-matched group were given ~93% of total restricted calories as high-fat and ~7% as regular chow. An additional group of age-matched mice was exposed to low-fat regular chow diet (#5001, Purina, St. Louis, MO, USA). Animal care and experimentation was approved by the Pennington Biomedical Research Center Institutional Animal Care and Use Committee and strictly followed rules and guidelines provided by the American Physiological Society and NIH.

A separate cohort of lean non-surgery male C57/BL6J mice at 10 weeks of age was purchased from Jackson Laboratories and fed a standard lab chow. Colons isolated from these animals were used in Ussing chamber studies.

### 2.2. RYGB Surgery

Details of our RYGB surgical procedure were reported previously [12]. Briefly, it consisted of a very small gastric pouch, a 5–6 cm long Roux-limb, a 4–5 cm long biliopancreatic limb, and a 10–12 cm long common limb.

### 2.3. Experimental Overview

After exposure to a high-fat diet for 12–14 weeks, mice weighed an average of 41.5 g and were randomly assigned to one of 3 groups: Sham-surgery (obese for the duration of the study), RYGB, and weight-matching to RYGB by calorie restriction. In addition, a fourth group of age-matched mice fed a low-fat diet without surgery was included in the same experimental protocol. Food intake was measured every 1–5 days and body composition every 2 weeks, and energy expenditure and locomotor activity were measured in metabolic chambers at 2–3 weeks and 8–9 weeks after surgery. At 12 weeks after surgery, mice were humanely killed, and tissues were harvested. Body weight, body composition, food intake, energy expenditure, and metabolic blood parameters were published in a separate paper [13].

### 2.4. Gene Expression Studies

Quantitative real-time reverse transcriptase PCR (RT-PCR) was used to assess the relative expression of nutrient GPCRs in human and mouse colonic tissues. Regional expression of the gastrointestinal (GI) appetite hormones PYY and glucagon (GCG), and TPH-1, an enzyme required for serotonin synthesis, was also determined. RNA was extracted from tissues using an RNeasy Mini kit (Qiagen, Hilden, Germany). RNA quantity and quality were assessed using a NanoDrop, and cDNA was obtained using a High-Capacity cDNA Reverse Transcription Kit with an RNase inhibitor. RT-PCR was performed using Taqman^®^ array cards pre-loaded with inventoried Taqman^®^ expression assays (Qiagen). Total target gene expression was determined relative to 18s expression, as previously described [1].

### 2.5. Immunohistochemistry

L-cells and EC cells were immunolabeled using antibodies against GLP-1 (1:200, SC-7782 Santa Cruz Biotechnology, Dallas, TX, USA) and 5-HT (1:400, ab66047 Abcam, Cambridge, UK), respectively. Neuronal innervation was assessed using pan-neuronal markers, protein gene product (PGP) 9.5 (1:800, 7863-0504 AbD Serotec), and the GLP-1 receptor (1:400, AGR-021 Alomone Labs). Staining for pERK (1:200, 4370 Cell Signalling, Danvers, MA, USA) and pCamKII (1:200, ab171095 Abcam) was performed following Ussing chamber experiments. Briefly, 10 μm sections were washed with blocking buffer. The primary antibody was applied (19 h, 4 °C), and tissues were washed with phosphate-buffered saline (PBS) and incubated (60 min, room temperature) with species-specific Alexa Fluor-conjugated secondary antibodies (1:200, Invitrogen, Carlsbad, CA, USA). A Leica DM4000 epifluorescence microscope was used to visualize immunoreactivity (IR) and images captured with a QImaging camera. Immunopositive cells for each group were manually counted in each section and averaged over five fields of view, as previously described [1]. Neuronal labeling was quantified using ImageJ software, where five fields of view (1.44 megapixel) were analyzed with ImageJ for the total number of immunoreactive pixels in the region of interest within the mucosal layer.

### 2.6. Ussing Chamber Experiments

As previously described, mouse colonic mucosae were divided into three 1 × 1 cm segments, and each segment was mounted in an Ussing flux chamber. The luminal surface was exposed to 10 mL nutrient solution, while the basolateral surface was exposed to 10 mL Krebs solution for 20 min. All solutions were carbogenated at 35–37 °C. Colonic mucosa was then fixed in 4% paraformaldehyde overnight.

### 2.7. Nutrient Solutions

The luminal side of the mouse colonic mucosa was exposed to a combination of GPR84 agonist lauric acid (25 mmol/L), GPR119 agonist PSN 375963 (10 μM, Tocris Bioscience, Bristol, UK), GPR40 agonist AS 2034178 (10 μM, Tocris Bioscience, UK) and TGR5 agonist TS-G 1005 (10 nM, Tocris Bioscience, UK). These were made up in Krebs solution: 124.05 mM NaCl (Sigma-Aldrich, UK), 4.78 mM KCl (AnalR UK), 1.33 mM NaH_2_PO_4_ (Sigma-Aldrich, UK), 2.44 mM MgSO_4_ (Sigma-Aldrich, UK), 2.50 CaCl_2_, 5.50 mM D-glucose (Sigma-Aldrich, UK), and 25.00 mM NaHCO_3_ (Sigma-Aldrich, UK) and carbogenated with 95% O_2_ and 5% CO_2_.

### 2.8. Statistical Analysis

All data are presented as mean ± SEM. The Student’s *t*-test was used for comparison between chow and sham groups. For comparisons between four study groups, one-way ANOVA was used, followed by Bonferroni-corrected multiple comparisons throughout. A *p* value of > 0.05 was considered significant.

## 3. Results

### 3.1. Obesity-Induced Up-Regulation of Colonic Nutrient-Sensing Receptors

**Figure 1:**
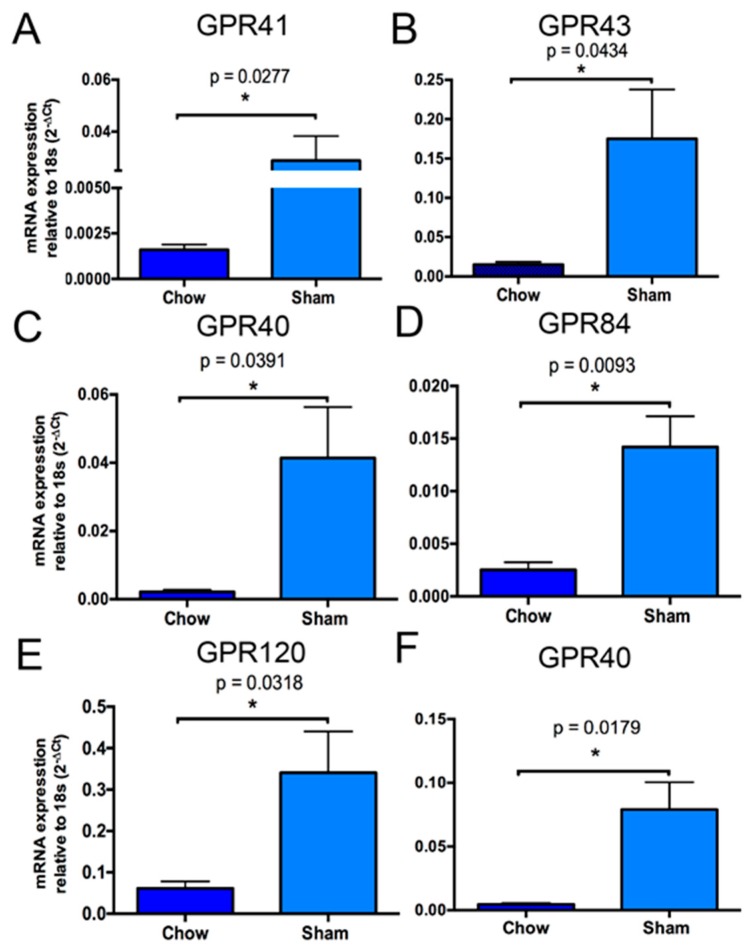

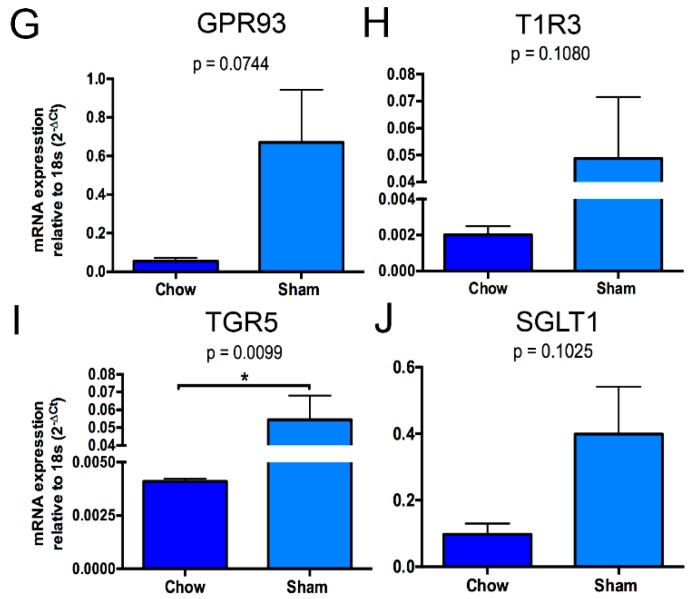
mRNA expression levels in chow (lean) vs. sham (obese) of short-chain fatty acid receptors GPR41 and GPR43 (**A**,**B**), medium-chain fatty acid receptors GPR40, GPR84 and GPR120 (**C**–**E**), lipid amide sensor GPR119 (**F**), amino acid-sensing receptors GPR93 and T1R3 (**G**,**H**), bile acid sensor TGR5 (**I**), and sodium-glucose co-transporter SGLT1 (**J**). The expression is described relative to 18s and represents N = 5–7. Error bars show SEM, and significance is *p* < 0.05 as denoted by *.

Colonic mRNA expression of nutrient-sensing receptors in obese mice was generally up-regulated compared to chow-fed lean mice. This up-regulation was statistically significant for short-chain fatty acid (SCFA) receptors GPR41 and GPR43, medium/long-chain fatty acid (M/LCFA) receptors GPR40, GPR84, and GPR120, lipid metabolite sensor GPR119, and bile acid-sensing receptor TGR5 (Figure 1). mRNA levels of T1R3, SGLT1, and GPR92 were not significantly different between chow and obese groups (Figure 1), although there was a trend towards increased expression, suggesting an overall increase in nutrient sensing.

### 3.2. RYGB Further Up-Regulates Expression of Specific Nutrient-Sensing Receptors

**Figure 2:**
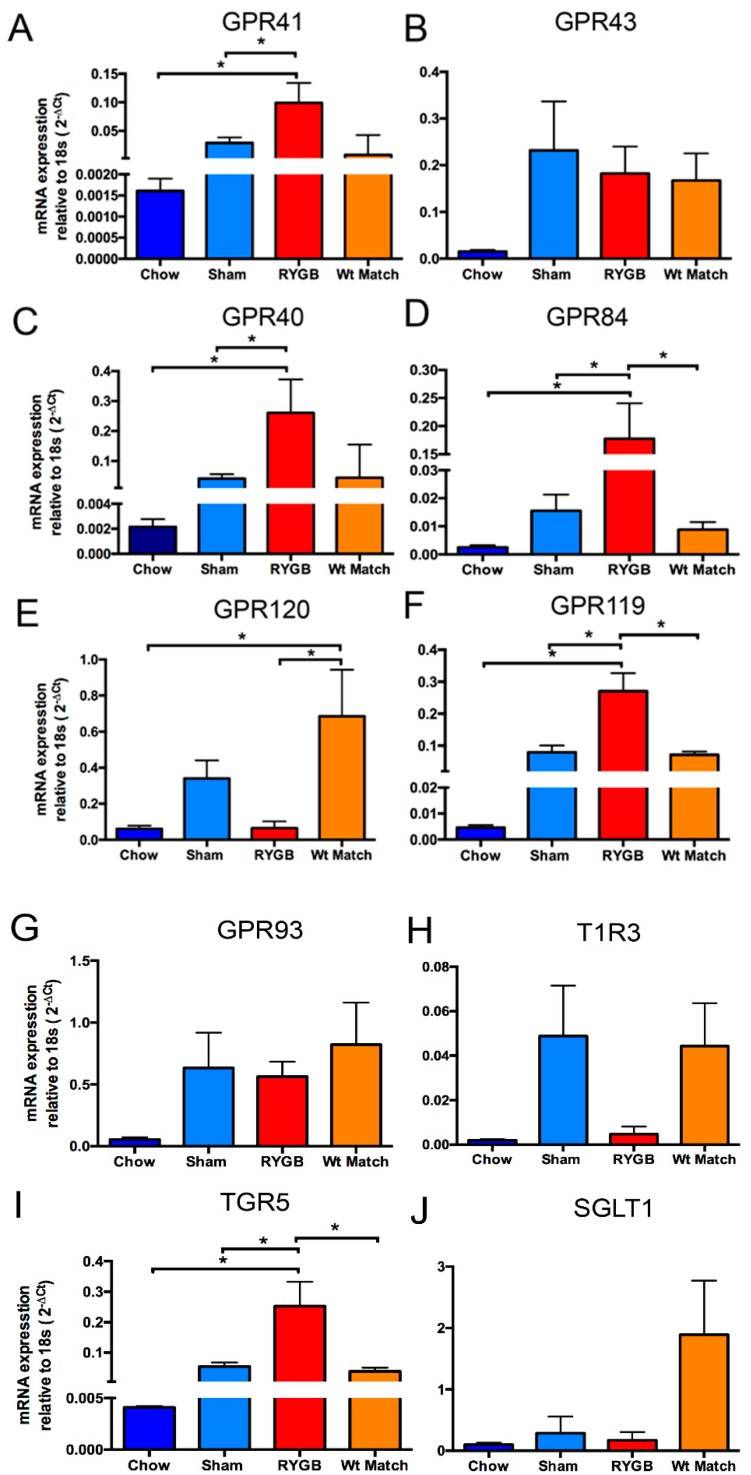
Altered colonic mRNA expression in obesity, Roux-en Y gastric bypass surgery (RYGB) of nutrient-sensing receptors for short-chain fatty acid receptors GPR41 and GPR43 (**A**,**B**), medium-chain fatty acid receptors GPR40, GPR84 and GPR120 (**C**–**E**), lipid amide sensor GPR119 (**F**), amino acid-sensing receptors GPR93 and T1R3 (**G**,**H**), bile acid sensor TGR5 (**I**), and sodium-glucose cotransporter SGLT1 (**J**). The expression is described relative to 18s and represents N = 5–7. Error bars show SEM, and significance is *p* < 0.05 as denoted by *.

Given the reduced food intake and body weight after RYGB, it is generally thought that it reverses obesity-induced impairment of intestinal nutrient sensing, but it is not clear whether this happens at the level of the nutrient-sensing receptor or downstream of the receptor. Our data show that most of the nutrient-sensing receptors are further up-regulated after RYGB. Importantly, this further up-regulation was specific to RYGB, but not for similar weight loss induced by calorie restriction. The expression of SCFA receptor GPR41 was significantly increased in the RYGB group compared to chow (lean) and sham operation (obese) (Figure 2A). However, mRNA expression of GPR43, another SCFA sensing receptor, increased in sham and remained elevated in RYGB and weight-match groups compared to chow groups. Interestingly, expression of MCFA receptor GPR40 significantly increased in the RYGB group compared to chow and sham groups, while GPR84 expression increased significantly compared to all groups (Figure 2C,D). Expression of GPR120, a sensor of omega-3 fatty acids, did not change after RYGB, but significantly increased in weight-matched controls compared to the RYGB and chow groups (Figure 2E). Importantly, expression of GPR119, which is expressed on L-cells, was significantly increased following RYGB compared to all other groups (Figure 2F). GPR119 releases GLP-1 following stimulation and is therefore important for insulin regulation in addition to appetite regulation. Similarly, bile acid receptor TGR5 expression was increased in RYGB compared to all other groups (Figure 2G). However, mRNA expression of the amino acid-sensing receptors GPR92/93 and T1R3 was not significantly altered in response to diet, RYGB or calorie restriction (Figure 2E,F). Similarly, expression of the glucose transporter SGLT1 was not altered (Figure 2G).

### 3.3. mRNA Expression of Appetite Modulation Hormones/Peptides Increases in Obesity and RYGB Both Increase Colonic L-Cell and EC Cell Numbers

**Figure 3:**
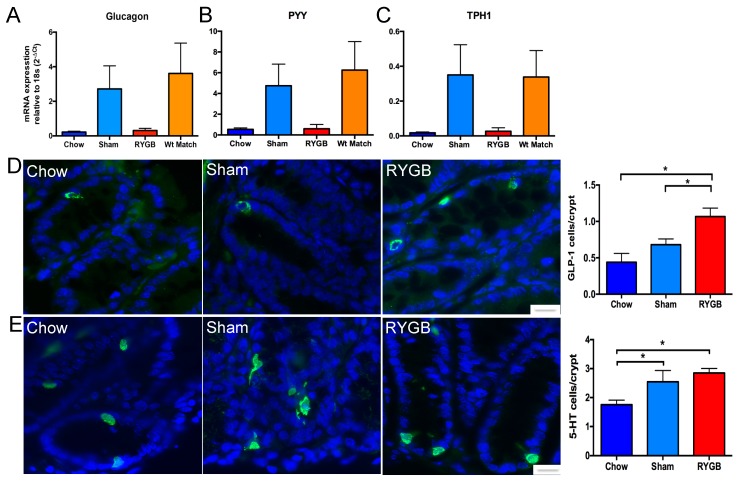
Colonic mRNA expressions of glucagon (the precursor to GLP-1), PYY, and TPH-1 (the enzyme required for 5-HT production) are elevated in sham and weight-match groups, but do not reach statistical significance (**A**–**C**). However, protein staining for GLP-1 show that RYGB significantly increases the number of GLP-1 containing L-cells compared to chow and sham groups (**D**). The number of 5-HT-containing EC cells increases in both sham and RYGB groups compared to chow groups (**E**). N = 5–7. Error bars show SEM, and significance is *p* < 0.05 as denoted by *. The scale bar = 5 μm.

mRNA expression of glucagon (the precursor to GLP-1 protein), PYY, and TPH-1 (the precursor enzyme critical for 5-HT production) was increased but did not reach statistical significance in the obese (sham-operated) group (Figure 3A–C). Expression levels decreased in response to RYGB to levels comparable to lean mice (Figure 3A–C). Protein expression was studied specifically with antibodies against GLP-1 to label L-cells and against 5-HT to label for EC cells in chow, sham-operated (obese), and RYGB groups. L-cells stained with GLP-1 showed no difference in numbers between lean and sham (obese) groups. However, there was a significant increase in RYGB mice compared to both sham-operated (obese) and chow controls (Figure 3D). Numbers of EC-cells increased as a result of obesity in the sham group compared to the chow group (Figure 3E). RYGB treatment appeared to further increase EC cells compared to the sham-operated (obese) group, although this did not reach statistical significance (Figure 3E).

### 3.4. Mucosal Innervation Is Unaltered by Diet and RYGB

**Figure 4:**
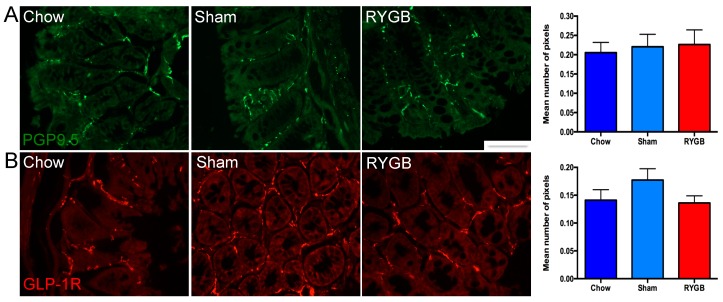
Expression of pan-neuronal marker PGP9.5 (green) is unchanged between chow, sham, and RYGB groups, as demonstrated by images showing consistent neuronal expression surrounding mucosal crypts (**A**). Quantification of PGP9.5 immunoreactivity (IR) in colonic mucosa showed no difference in the number of positive pixels between the three groups (**B**). GLP-1 receptor expression was found in close apposition to the basal end of colonocytes (red), and quantification of pixels showed no change in IR between the groups. N = 5–7. The error bars show SEM, and the scale bar = 10 μm.

Since changes were observed in the sensory apparatus in terms of expression of nutrient-sensing receptors and EECs, we wanted to know if downstream elements in nutrient sensing also adapted after diet and RYGB intervention. We used the pan-neuronal marker PGP9.5 to explore overall neuronal density in the colonic epithelium and found that there was no change in the number of PGP9.5-positive pixels between chow, sham-operated, and RYGB groups, with consistent patterns of neuronal staining between colonic crypts observed (Figure 4A). We then looked at GLP-1R to see if an altered luminal environment could specifically affect the mucosal expression of this receptor, which is known to be localized on vagal afferents [14]. Positive staining was observed around crypts and in close apposition to the basal side of colonocytes. However, image analysis revealed no differences in pixel number or expression patterns between the three groups (Figure 4B).

### 3.5. Mucosal Stimulation with Specific Nutrients Activates Colonocytes

**Figure 5:**
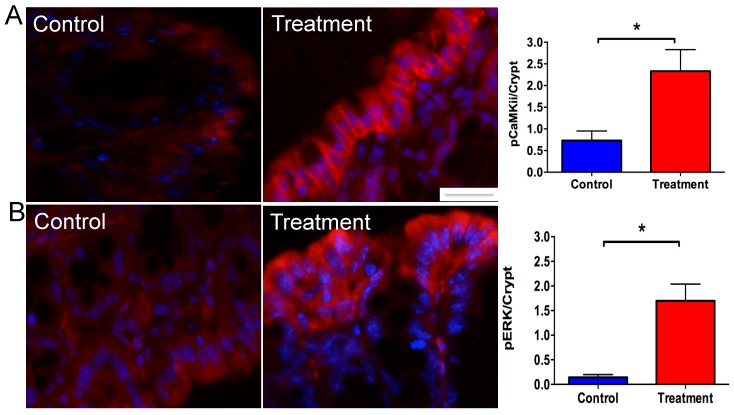
Stimulation of colonic mucosa with GPR84 agonist lauric acid (25 mmol/L), GPR119 agonist PSN 375963 (10 μM), GPR40 agonist AS 2034178 (10 μM), and TGR5 agonist TS-G 1005 (10 nM) activates both pERK and pCamKII intracellular pathways, as shown by a significant increase in the number of IR cells (**A**,**B**). Both pERK and pCamKII IR cells were commonly observed to be the most superficial cells, with cells within crypts sparsely stained (**A**,**B**). N = 3. The error bars show SEM, and significance is *p* < 0.05 as denoted by *. The scale bar = 10 μm.

Data from the mRNA expression studies revealed that specific nutrient-sensing GPCRs were upregulated in obesity and further increased after RYGB treatment. Since these GPCRs are involved in EEC and EC activation, it follows that they may be involved in mechanisms reducing appetite and food intake by activating, for example, L-cells and EC cells, thus enabling release of anorectic hormones/peptides. We specifically chose GPR119, GPR84, GPR40, and TGR5 to use as a combined stimulus since these receptors were up-regulated in obesity and RYGB, and as GPR119 and GPR40 have been shown to exhibit synergistic effects on GLP-1 secretion [15]. Following 20 min of stimulation with combined agonists, the number of pERK and pCamKII-expressing cells significantly increased (Figure 5A,B), confirming the functional validity of the targets we chose for molecular analysis in the main part of this study.

## 4. Discussion

Our data show that nutrient-sensing GPCRs respond in specific patterns to obesity and RYGB. Somewhat unexpectedly, there was a strong general trend for nutrient-sensing receptor expression to be up-regulated in the high-fat-fed obese (sham-operated) vs. chow-fed lean mice, although this did not reach statistical significance for some GPCRs. This was unexpected because there is considerable support for the notion that nutrient-sensing mechanisms are blunted or impaired in obesity [16,17]. One explanation for up- rather than down-regulation of nutrient receptors is that, at least in the colon, the system adapts to blunted nutrient sensing by up-regulation at the nutrient receptor mRNA level, and that obesity-induced impairment of the colonic nutrient-sensing signal must be further downstream. There are no other studies on expression levels of nutrient receptors in the colon, but a study in overweight and obese humans shows that GPR40, GPR120, and GPR119 mRNA expression levels in the duodenum are unchanged compared to lean controls [18]. Therefore, changes are more distal or are only evoked by a specific dietary intervention, such as in a high-fat diet.

Additional analyses will be necessary to investigate receptor coupling to hormone secretion and vagal afferent detection in the colon, and to extend our findings to the upper parts of the GI tract.

Because RYGB almost completely reverses obesity, it could also be expected to reverse increased expression of nutrient-sensing receptors associated with obesity. This was the case for long-chain fatty acid receptor GPR120, which RYGB reduced to the level of chow-fed lean controls. The fact that this reversal was only seen after RYGB but not weight-matching suggests that it is mediated by a weight-loss-specific effect, independent of the surgery. However, this was clearly not the case for GPR 41, 40, 84, and 119, which were further enhanced 12 weeks after RYGB as compared to the sham-surgery group, again suggesting that increased expression is caused by the high-fat diet rather than the obese state.

The observation that expression of MCFAs, lipid sensors, and bile acid-sensing receptors changes following RYGB suggests that exposure of previously naïve GPCRs to their nutrient agonists in the colon promotes transcription. Increased mRNA expression may result in increased protein expression of receptors on the surface of EECs and EC cells, which could increase cell activation and hormone/peptide release. Several studies have shown that GPCR activation can indeed promote activation of these cells. Examples of GPCR activation include stimulation of GPR40 by small molecule agonists which promote L-cell activity via release of GLP-1 in diet-induced obese mice [19], GPR84 activation via endogenous ligand lauric acid releasing GLP-1, PYY, and 5-HT in stimulated human colonic tissue (ex vivo) [1], synthetic GPR119 agonist increasing GLP-1 levels in L-cells [20] and obese mice [21], and GPR43 agonists increasing GLP-1 secretion from human cecum (NCI-H716 cells) [22] and primary mouse colonic cultures [23].

We also found mRNA expression of SCFA sensor GPR41 increased after RYGB compared to the obese group, suggesting that an altered luminal environment can also drive molecular changes. Colonic mRNA expression of both GPR41 and GPR43 is raised following obesity, as we and others have shown [24]. RYGB alters the gut microbiome in rats, mice, and humans [25,26], and favors the growth of Gammaproteobacteria (Escherichia) and Verrucomicrobia (Akkermansia) [26], which is likely to cause changes to SCFA receptors, especially since *Akkermansia municiphilla* is associated with propionate production [27]. Both GPR41 and GPR43 are required for SCFA sensing. However, no change in GPR43 expression was observed after RYGB, although we did find increased expression in obesity, as others have described [24]. Protein expression levels may reveal changes, and therefore, we cannot at this stage conclude that GPR43 expression is not required for RYGB-induced weight loss.

Our finding of elevated TGR5 expression specifically after RYGB, but not after calorie restriction-induced weight loss, is in agreement with other studies that have shown increased circulating bile acid levels after bariatric surgery [28]. This finding is likely to be a factor in increased GLP-1 secretion by TGR5 stimulation [29] after RYGB. However, we have recently demonstrated that TGR5 signaling is not required for any beneficial effects of RYGB in mice [30], suggesting that bile acid signaling through FXR may be more important [31].

It is likely that EEC and EC cells express a range of nutrient-sensing GPCRs at any given time. The colon has amongst the largest density of EEC and EC cells [32] in the GIT. However, compared to the number of colonocytes, the number of hormone/peptide releasing cells is low. Therefore, it is plausible that these cells need to express on their cell surface a variety of receptors to effectively respond to the variety of nutrient components found in diets. Our use of multiple GPCR agonist stimulation was to test the capability of normal mouse colonocytes to respond to agonists of GPCRs that were elevated in the RYGB group. We found that the majority of superficial colonocytes, most highly exposed to stimulation in Ussing chambers, were activated as demonstrated by pERK and pCamKii staining. Our data suggest further studies using GPCR agonist stimulation may be able to recapitulate the effects of RYGB by activating EEC and EC cells.

Increased expression of both L-cells and EC cells has been described in rat models of RYGB. We and others have shown that hyperplasia resulting from RYGB increases the number but not the density of EECs (L-cells, I-cells, and N-cells) and EC cells in the Roux and common limbs [33], alimentary channel [34], and terminal ileum [35]. Data from this study showing L-cell numbers increase in the colon following RYGB are consistent with these findings. We also found a trend for increased L-cell numbers in colons of obese vs. lean mice, although statistical significance was not reached. Nevertheless, these data follow the trend of increased L-cell density following lipid-rich diets in both humans and mice [36]. Therefore, the plasticity we report in nutrient-sensing GPCRs expression may drive not only enhanced cell activation, but also cell differentiation. Colonic EC cell numbers were significantly increased in the obese group of this study, confirming increased 5-HT content and TPH1/2 mRNA expression in long-term diet-induced obesity (DIO) mice [37]. We do not see any further increase in EC cell number following RYGB, suggesting that obesity induces maximal EC cell differentiation. However, it is clear that nutrient load and content have a significant influence on EEC and EC cell density, which may be attributed to specific GPCRs (and therefore specific nutrients) which can optimally enhance EEC and EC cell numbers and hormone/peptide release.

Our finding that overall innervation of the colonic mucosa is unchanged in obesity and RYGB reflects what has been demonstrated for vagal afferent innervation after RYGB in other parts of the GIT [38]. Unchanged GLP-1R expression in colonic mucosa agrees with our previous work as well as others showing GLP-1R is not required for the beneficial effects of RYGB [39,40]. However, GLP-1 acting on GLP-1R expressed on pancreatic β-cells is required for partial improvement of glucose tolerance after RYGB in mice [41].

Overall, our study demonstrates for the first time that expression of nutrient receptors, and numbers of GLP-1 containing L-cells and 5-HT cells, are significantly and specifically affected by high-fat, diet-induced obesity and RYGB. In particular, we report that expression of specific nutrient-sensing GPCRs is up-regulated by both obesity and even more robustly by RYGB, suggesting that these receptors may drive EEC and EC cell differentiation, as well as activation. Indeed, we confirmed that stimulation with agonists (for these GPCRs) does cause cell activation, suggesting these receptors may be a non-surgical means of enhancing the release of anorectic hormones/peptides.
